# Progress in Rett Syndrome: from discovery to clinical trials

**DOI:** 10.1007/s10354-016-0491-9

**Published:** 2016-08-04

**Authors:** Alan K. Percy

**Affiliations:** Civitan International Research Center, University of Alabama at Birmingham, 1720 2nd Avenue South, 35294-0021 Birmingham, AL USA

**Keywords:** Andreas Rett, Rett syndrome, MECP2, Natural history study, Clinical trials, Andreas Rett, Rett-Syndrom, MECP2, Verlaufsstudie, Klinische Studien

## Abstract

Fifty years ago, Andreas Rett described a disorder in 22 females featuring prominent regression of fine motor and communication skills, cognitive impairment, stereotypic movements, periodic breathing, and gait abnormalities. This disorder became known as Rett syndrome (RTT) following the report of Hagberg et al. in 1983. Although RTT was scarcely recognized at that time in the United States, here the efforts of Rett and Hagberg led to rapid progress in recognition and diagnosis, a clearer understanding of its clinical and pathological underpinnings, and, ultimately, identification of mutations in the methyl-CpG-binding protein 2 (*MECP2*) gene as the primary cause of this unique and challenging neurodevelopmental disorder. Thereafter, a natural history study and critical translational research in animal models paved the way for potential disease-modifying agents to be assessed in human clinical trials. To be successful, the energies of the international community at all levels, including researchers in clinical and basic science, funding agencies, pharmaceutical companies, patient advocates, and, above all, parents and their children are essential. Otherwise, hopes for effective treatment, if not, a cure, will remain unfulfilled.

## Zusammenfassung

Vor genau 50 Jahren beschrieb Andreas Rett eine Erkrankung bei 22 Mädchen mit auffallender Regression feinmotorischer und kommunikativer Fähigkeiten, kognitiven Defiziten, stereotypen Bewegungsmustern, periodischer Atmung und Gangauffälligkeiten. Diese Erkrankung wurde nach einer Veröffentlichung durch Hagberg et al. 1983 als Rett-Syndrom bekannt. Obwohl das Syndrom zu jener Zeit in den USA kaum bekannt war, führten die Leistungen von Rett und Hagberg rasch zu vermehrter Erkennung und Diagnosestellung, einem klareren Verständnis der klinischen und pathologischen Grundlagen und schlussendlich der Identifikation von Mutationen im Methyl-CpG-binding-Protein-2(*MECP2*)-Gen als primärer Ursache dieser besonderen, herausfordernden neurologischen Entwicklungsstörung. Danach ebneten eine Longitudinalstudie zum natürlichen Krankheitsverlauf und entscheidende Untersuchungen am Mausmodell den Weg für die Erforschung potenziell krankheitsmodulierender Substanzen in klinischen Studien. Für die Aussicht auf Erfolg ist der Zusammenschluss aller Energien auf internationaler Ebene wichtig – unter Einbezug der Wissenschaftler in Klinik und Grundlagenforschung, der Forschungsförderung, der pharmazeutischen Industrie, von Patientenvertretern und vor allem von Eltern und betroffenen Kindern. Andernfalls wird der Wunsch nach effektiver Behandlung, wenn eine Heilung schon nicht möglich ist, nicht in Erfüllung gehen.

## Introduction

In 1965, Andreas Rett, a neuropediatrician in Vienna, published the first report of a neurodevelopment disorder involving females with early onset of developmental delay followed by frank regression, loss of communication and fine motor skills, and the appearance of stereotypic hand movements and periodic breathing during wakefulness [1]. Dr. Rett attempted to ascertain and promote recognition of similar features by other physicians throughout central Europe with little success. Around the same time, Bengt Hagberg, a child neurologist in Uppsala, was recognizing the same constellation of clinical characteristics in females from throughout Sweden, although these two clinicians did not meet directly for another 15–20 years. In a series of laboratory studies looking for a metabolic marker for this disorder, Rett had reported elevated blood ammonia levels and published these results in 1977 in the *Handbook of Clinical Neurology* related to hyperammonemias [2]. During this intervening period, Hagberg was discussing this disorder with other child neurologists throughout Europe, recognizing the clinical similarities, but also failing to note the same blood ammonia findings as described by Rett. At that point and with the enthusiastic participation of Jean Aicardi, Karin Dias, and Ovidio Ramos, Hagberg began to prepare a paper on 35 females with this disorder from Sweden, France, and Portugal. However, when he became aware that blood ammonia results identified in Vienna were spurious, and following a chance meeting with Rett in Toronto in 1981, Hagberg decided to call this disorder “Rett syndrome”. The paper appeared in the *Annals of Neurology* in 1983, and suddenly child neurologists, neuropediatricians, and geneticists around the world were alerted to the existence of this new and unique neurodevelopmental disorder [3]. Hagberg continued to reveal additional findings, including developing a staging system for RTT [4], identifying variant forms of RTT [5], and leading the way first with initial criteria development in 1984 [6] and then with their revision in 2002, following the identification of mutations in *MECP2 *[7].

In the United States, scarcely anyone knew about this disorder prior to the Hagberg et al. paper [3]. Mary Coleman, a child neurologist in Washington DC, had attended a meeting in Paris and learned of Rett syndrome (RTT). Vanja Holm, a neuropediatrician from Seattle, learned of this disorder on a visit to Sweden. For myself, a pediatrician in Houston, who suspected the disorder based on the *Annals of Neurology* paper, referred a girl to the child development clinic at Texas Children’s Hospital where I was the child neurologist. The diagnosis of RTT was confirmed on examination of this young female and, following a meeting with other child neurologists at Baylor, five additional girls with RTT were identified within the next few months. As the result of questions raised by the parents of the first girl, contact was made with both Bengt Hagberg and Andreas Rett, and an invitation was received to attend the RTT meeting in Vienna in 1984. Dr. Holm and Dr. Hugo Moser from the Kennedy Krieger Institute (KKI) also attended. This international meeting provided remarkable stimulus for the expansion of studies on RTT throughout the world. Subsequent meetings in 1986 and 1988 served to expand clinical, laboratory, and pathological studies attempting to elucidate metabolic and genetic signals characteristic of RTT. Importantly, the 1984 meeting provided the opportunity for development of diagnostic criteria (see above) that were critical to the field going forward.

## Initial developments in the US

In 1984, a RTT clinic was formed at Baylor together with Daniel Glaze, Huda Zoghbi, and Dawn Armstrong, and other clinics emerged at the KKI in Baltimore, in San Diego, and in Portland (Oregon). At the same time, the International Rett Syndrome Association (IRSA), a vital patient advocacy group, was created by Kathy Hunter, Gail Smith, and Jane Brubaker. This proved critical from several perspectives. First, IRSA resulted in a focal point for parents, other caregivers, and interested physicians to meet. Second, Andreas Rett was invited to visit the US on several occasions to provide consultation with physicians and to examine and confirm diagnoses in affected individuals. Third, Congressman Steny Hoyer from Maryland was sufficiently moved by the situation to promote legislation providing for research funds from the National Institutes of Health (NIH). With that, program projects were awarded to Baylor and to KKI, markedly increasing research activities.

Among the initial reports that emerged were the recognition of abnormalities in biogenic amine metabolites in cerebrospinal fluid [8], neurophysiologic abnormalities related to seizures [9] and periodic breathing [10], and pathological investigations led by Dr. Armstrong that revealed the brain to be smaller than normal, to have reduced melanin pigmentation in substantia nigra and other pigmented regions, and, at the microscopic level, a reduction in neuronal size and synaptic complexity with specific alterations in dendrites [11–18]. What was not found was evidence of a progressive neurodegenerative process. This last feature suggested that RTT was a neurodevelopmental abnormality that could be potentially reversible with the proper strategies.

The etiology of RTT was considered by many to be genetic, based on the exclusive occurrence, at least at that time, of RTT in females, suggesting an X‑linked, dominant disorder. However, the efforts to identify a biochemical or metabolic fingerprint greatly hampered further understanding. At Baylor, Dr. Zoghbi was then engaged in training in molecular genetics and was encouraged to pursue studies into a molecular etiology for RTT. Interestingly, the presence of an autosome–X chromosome translocation in one of the first children evaluated allowed a large portion of the X‑chromosome to be excluded [19]. Gradually, the area of interest was narrowed to Xq28 by a series of studies from the rare instances of familial involvement [20, 21]. In this regard, the general failure of recurrence in families and the X‑linked dominant nature suggested that a mutation, if one could be identified, would result from a germline de novo mutation in the father [22]. Although this idea was questioned by some [23], the molecular search continued and in 1999 a mutation in the methyl-CpG-binding protein 2 (*MECP2*) gene, located at Xq28, was identified by Ruthie Amir [24] working in the Zoghbi laboratory. Subsequently, these findings were confirmed in multiple laboratories throughout the world [25–28]. Recognition of males with RTT and both females and males with *MECP2* mutations were now confirmed [29, 30]. Males with RTT were the result of the co-occurrence of Klinefelter syndrome (47XXY) [31–34] or somatic mosaicism [35]. Familial occurrences were associated with maternal mutations in which the transmitting female was either normal or had mild learning difficulties or cognitive impairment [36–39]. Further on, with this new information a number of laboratories began to examine the role of *MECP2* in brain development and function. Interestingly, *MECP2* had been well-described in tumor biology for more than 10 years prior to this linkage with RTT, due to its possible relationship to methyl-binding domains in DNA [40–50]. Laboratory studies have persisted and expanded over the years to include potential disease-altering therapies and possible genetic intervention to provide a more fundamental curative avenue. In this regard, the studies of Guy et al. in a mutant mouse indeed showed that if a normal gene were in place, near-normal recovery of function was possible [51].

At the same time, efforts in support of research into the clinical and laboratory bases for rare diseases had been enabled at the NIH through congressional mandate. As a result, the Rare Disease Clinical Research Network was formed and initial funding became available in the early 2000s. This resulted in the funding of a natural history study (NHS) regarding RTT that has been refunded twice and now, in its third iteration, is addressing RTT, *MECP2* duplication disorders, and other RTT-related disorders including *CDKL5*, *FOXGL*, and individuals with *MECP2* mutations, both females and males, who do not meet the diagnostic criteria for RTT. To date, data from more than 1200 participants evaluated in the first 11 years of the NHS have been published and several additional topics are being analyzed for submission. The current NHS study is continuing to gather information on the natural history of the above mentioned disorders, collecting biologic samples for possible detection of a biomarker, and investigating the event-related potentials of hearing and vision (auditory brainstem and visual evoked potentials). One of the initial outcomes of the NHS was validation of the revised consensus criteria of 2010 following the convening of an international panel of clinicians [52, 53]. These modifications simplified the criteria and provided a distinction for their application in classic and variant or atypical RTT (Table 1).

**Table 1:** Consensus criteria for classic and variant Rett syndrome (RTT) [52]

*Requirement for classic or typical RTT*	A period of regression followed by stabilization and recovery: 1. All main and all exclusion criteria 2. Supportive criteria not required although may be present
*Requirement for variant or atypical RTT*	1. A period of regression followed by stabilization and recovery 2. At least 2 of 4 main criteria and 5 of 11 supportive criteria
*Main criteria*	1. Partial or complete loss of acquired purposeful hand skills 2. Partial or complete loss of spoken language 3. Dyspraxic gait or inability to ambulate 4. Stereotypic hand movements: hand mouthing, hand wringing/clasping, hand clapping, or finger rubbing
*Exclusion criteria*	1. Brain injury: peri- or postnatal trauma, neurometabolic disease, or severe infection involving neurological function 2. Grossly abnormal psychomotor development in first 6 months after birth
*Supportive criteria for variant RTT*	1. Periodic breathing during wakefulness 2. Bruxism while awake 3. Altered sleep pattern 4. Abnormal muscle tone 5. Peripheral vasomotor disturbance 6. Scoliosis/kyphosis 7. Growth failure 8. Small cool/cold hands and/or feet 9. Inappropriate laughing or screaming spells 10. Delayed or diminished response to pain 11. Intense eye communication or “eye pointing”

## A multisystem problem

Despite the primary involvement of the central nervous system (CNS), RTT is a multisystem problem. In addition to brain growth, stereotypies, epilepsy, periodic breathing, sleep, and other problems directly related to the CNS, multiple other systems are affected, including growth and nutrition, the gastrointestinal tract, and pubertal development. Overall, longevity is reduced by about one third compared to typically developing females. These aspects are discussed individually below, along with other important clinical features.

## Developmental skills

The acquisition of developmental skills was long suspected of being abnormal, although specific data had been lacking. As such, early development had been characterized as “apparently” normal. Beginning with the initial studies of Einspieler and Marschik taking advantage of video recordings during infancy, substantial deviations from normal were noted [54–60]. Data from the NHS confirmed these findings and extended them to include individuals with both classic and atypical or variant RTT [61]. Longitudinal assessments in the NHS allowed careful analysis of development over time. Early developmental skills are generally acquired, but many of these skills are acquired later than normal by most. Gross motor and receptive language acquisition is much better than fine motor and expressive language skills. The more complex motor and communication skills such as managing stairs, riding a tricycle, or demonstrating facility with reading or mathematics are delayed or absent. Clinical severity is less in those acquiring more skills. Better outcomes are associated with specific mutations, as described below.

## Epilepsy

The electroencephalogram (EEG) pattern is generally abnormal by age 3 years, with slowing of normal background and epileptiform wave patterns, often worse during sleep. Seizures may be focal, generalized, or atypical absence, and will occur in more than 85 % by age 16 [62]. However, the frequency of seizures at any given age is highly variable, requiring medication for appropriate management in 35–40 %. Video-EEG monitoring is often required for differentiation of seizures from nonepileptic behaviors. Typically, the EEG becomes less epileptiform after age 20 and seizures may abate significantly. Conversely, premenstrual seizures (catamenial epilepsy) may be an issue that requires chronic medication or short-term coverage with a long-acting benzodiazepine such as clonazepam.

## Genotype–phenotype correlation

Definite genotype–phenotype correlations are noted with important caveats [63–65]. For both classic and atypical RTT, R133C, R294X, R306C, and 3’-truncations result in less severity than other common mutations (R106W, R168X, R255X, R270X, deletions/insertions, and splice site mutations). Overall, clinical severity tends to increase slowly with age as the result of scoliosis, dystonia, and rigidity. Those who ambulate, maintain some hand function, and have a later age at onset also tend to be less severely impacted.

However, markedly different clinical patterns or outcomes can be seen in two females with exactly the same genotype. These are most likely related to differences in X‑chromosome inactivation (XCI), differing genetic backgrounds, environmental factors, and the clonal distribution of normal and abnormal X‑chromosomes throughout the brain. From a relatively small study in blood involving 183 participants in the NHS (Friez et al., unpublished work), significant skewing of XCI was noted, including 11 % that were highly skewed and 26 % were moderately skewed. Overall, 51 % were random and 12 % were uninformative. Of the 11 % who were highly skewed, this was evenly divided between the normal and the abnormal X‑chromosome and, thus, conferred an exclusive advantage in neither direction.

## Age at diagnosis

Considering the increasing potential for clinical trials, earlier identification and diagnosis is regarded as essential. Initially, the average age at diagnosis was found to be greater than 4 years, but over the course of the NHS, the average age has fallen, now being 2.7 years [66]. In the US, the overwhelming percentage (90 %) of diagnoses are made by child neurologists, neuropediatricians, or geneticists. Regression was noted in 90 % between age 12 years and 30 months. To increase diagnoses by primary care physicians, certain clues can lead directly to diagnosis or to referral to a subspecialist. These include declining head circumference in early infancy, declining weight and height percentiles, slowing or plateauing of development or frank regression, and the child appears to the parents to be “a really good baby”, that is, the infant is “too good”. Greater attention to these points could accelerate diagnosis.

## Growth

Declining growth rates are typical [67–69]. First is the declining head circumference growth rate, which may be seen as early as 1.5 months with the median value reaching the 2^nd^ percentile by age 2 years. Weight, as early as 6 months, and height, as early as 17 months, follow, with the median values reaching the 2^nd^ percentile by age 12 years [69]. Hands and feet are also small, with the feet being relatively smaller [68].

## Gastrointestinal features

Problems involving the gastrointestinal tract are seen from top to bottom [70–73]. These include manipulation of food in the mouth, swallowing issues, gastroesophageal reflux, delayed gastric emptying, constipation, and gallbladder dysfunction. Food manipulation in the mouth manifesting as prolonged feeding times, gastroesophageal (G–E) reflux, and constipation are seen in the majority. Gallbladder dysfunction is seen in about 3 %, but has been seen as early as age 2–3 years. Given the inadequate ability to describe pain, these issues deserve primary attention when the individual is upset or unhappy, or awakens frequently in the night. Gastrostomy tube placement provides some or exclusive feeding in >30 % [70].

## Scoliosis

Scoliosis is present in the overwhelming majority of girls by age 16 and in 8 % of preschoolers, that is by age 4 or earlier [74, 75]. Progression should cease at maturity, but may increase slowly thereafter. Varying degrees of severity are noted, usually by age 8. The curvature is typically greater in the child who is non-ambulatory and who has significant truncal hypotonia. Although no formal study has been done in RTT, bracing is often considered with curves greater than 25^º^. Surgery is recommended when the curve exceeds 40^º^. In the NHS, 13 % had surgery. Most parents agreed that the quality of life was improved, both for the individual and the family [75].

## Prolonged QTc

Prolongation of the QTc interval is present in approximately 20 % of individuals in the NHS [76, 77]. While this is generally in the borderline range, occasionally it is sufficiently elevated to warrant treatment by β‑blockers. Rarely, QTc levels exceed 600 ms, in which cases a pacemaker has been required. McCauley et al. [77] demonstrated similar QTc prolongation in an animal model of RTT and found that sodium channel blockade was more effective than β‑blockers. However, this has not been studied in humans.

## Puberty and menarche

Puberty and menarche generally occur similar to typically developing females, but important differences do exist [78]. Onset of puberty is premature in 25 %, i.e., occurring before age 8 years, but the interval between onset of puberty and menarche is longer than typical: 3.9 years vs. 3.0 years in typically developing females. The median age for pubertal onset is shorter for breast development and longer for pubic development by about 6 months. Conversely, the age at menarche is later than normal, age 13.0 vs. 12.5 years. The earlier onset of puberty is correlated with increased body mass index and the so-called milder mutations.

## QOL for affected and caregivers

Quality of life (QOL) is an important component of care for individuals with chronic disease and has proven equally critical in RTT. Examining both cross-sectional and longitudinal results from individuals with RTT and their primary caregiver, typically the mother, revealed that poor motor function is associated with greater clinical severity [79]. However, poor motor function resulted in fewer behavioral problems and conversely, better motor function resulted in more behavioral issues. For example, those who were ambulatory could climb on furniture, open doors and leave the home, or injure themselves near a stove. This raises the potential concern that modest improvement in motor function could result in greater behavioral issues [79].

From the caregiver’s perspective, as the parents age and may develop problems of their own, their physical QOL declines, whereas mental QOL tends to improve [80]. This is related to achieving an accommodation with the issues associated with RTT and to the decline in some interfering or concerning patterns such as bruxism, periodic breathing, and general difficulties in management. However, it is noted that once these individuals age out of school, the availability of adult programs may be significantly limited and will impact the overall caregiving.

## Survival

An initial analysis involving the NHS and the IRSA registry demonstrated that survival in individuals with RTT was normal through the first 10 years, but declined significantly thereafter, with >75 % alive at age 30 and >50 % at age 50, the latter being significantly less than normal survival in US females [81]. However, when compared with the original group described by Andreas Rett, overall longevity had at least doubled [82]. This is related to improved diagnosis, nutrition, physical and occupational therapies, and overall management strategies.

## Changing pattern of survival

Subsequently, a more recent examination of survival and causation of death in the NHS confirmed the average survival beyond age 50, showing directly that preserved ambulation or assisted walking, maintenance of adequate weight, and effective seizure control promoted survival [83]. The extreme frailty reported in the study of Kerr et al. in the 1990s was not seen in the NHS in classic RTT. Death was often unwitnessed and causation was difficult to determine, but cardiorespiratory issues were regarded as the most likely cause of difficulties. These results emphasize the positive effects of diet and effective therapies.

## Clinical trials

A number of clinical trials have been conducted, beginning already in the era before the identification of mutations in *MECP2*. The opiate antagonist naltrexone was assessed as a treatment for periodic breathing. No benefit was noted, but the design of the trial did not account for differing clinical severity between the treatment and placebo groups, and could have confounded the results [84]. However, it was shown in a parallel study that the intravenous antagonist, naloxone, resulted in EEG slowing. Any beneficial effects could have resulted merely from sedation. With the identification of mutations in *MECP2 *and the potential role of DNA methylation, a trial of folate-betaine was conducted in the early 2000s. Despite parent reports of improvement, no positive objective evidence was noted [85].

More recently, clinical trials have been conducted with IGF-1 at Boston Children’s Hospital and with NNZ-2566 (trofinetide), the terminal tripeptide of IGF-1, at Baylor College of Medicine, UAB, and Gillette Children’s Hospital. Both agents proved to have no significant safety or tolerability issues. The IGF-1 trial, based on a translational research study [86], produced evidence of safety [87] and is continuing at multiple sites. The NNZ-2566 trial in older girls and women provided preliminary evidence of efficacy and a second phase 2 trial is being conducted in children aged 5–15 [88].

Other trials are in early stages of development or enrollment. In a multisite trial, sarizotan, a serotonin and dopamine receptor agonist, will be tested in girls and women aged 13–50, and a trial with ketamine is now starting at the Cleveland Clinic [89]. Both agents are targeting the improvement of periodic breathing.

## Future perspectives

In the more than 30 years that I have been involved with Rett syndrome (RTT), remarkable progress has been accomplished both clinically and in the laboratory. Improved management of individuals with RTT and effective guidance not only in terms of medical problems, but also with regard to proper nutrition, therapeutic interventions, and the new and emerging field of augmentative communication using computer-assisted strategies, have been beneficial. Taking the broader perspective for progress in RTT, the energies of the international community at all levels of research, from basic through translational to clinical studies, are required. Many disease-modifying strategies are advancing through translational research and reaching the level of clinical trials. At the same time, strategies to reverse the underlying genetic defect are continuing, whether to replace the defective gene or reverse X‑chromosome inactivation. These approaches do offer fundamental challenges that must be overcome. The expansion of clinical trials will require efforts of all, including the commitment of funding agencies, pharmaceutical companies, researchers, patient advocacy groups, and families at the very least. Throughout the world, new and more investigators must be recruited. Parent advocacy groups must continue to press for continued progress. Clinical trials are essential, but are also labor intensive, demand careful conduct, and require direct participation of families and other caregivers. They require patience and courage, focus and understanding. Above all, human involvement is a prerequisite. Without the involvement of everyone, the desired goals of effective treatment and, ultimately, a cure, are an unachievable illusion.
